# The Causes and Consequences of DNA Damage and Chromosomal Instability Induced by Human Papillomavirus

**DOI:** 10.3390/cancers16091662

**Published:** 2024-04-25

**Authors:** Kathryn M. Jones, Ava Bryan, Emily McCunn, Pate E. Lantz, Hunter Blalock, Isabel C. Ojeda, Kavi Mehta, Pippa F. Cosper

**Affiliations:** 1 Department of Human Oncology, University of Wisconsin, Madison, WI 53705, USA; 2 University of Wisconsin School of Medicine and Public Health, University of Wisconsin, Madison, WI 53705, USA; 3 Department of Comparative Biosciences, University of Wisconsin, Madison, WI 53705, USA; 4 Carbone Cancer Center, University of Wisconsin, Madison, WI 53705, USA

**Keywords:** human papillomavirus (HPV), chromosomal instability (CIN), DNA damage response, radiation, alternative end-joining, mitosis

## Abstract

**Simple Summary:**

Human papillomavirus (HPV) causes 5% of cancers and is the main cause of oropharyngeal cancer in the United States and of cervical cancer worldwide. HPV proteins induce DNA damage and exploit and hijack the host DNA damage response. The HPV oncoproteins E6 and E7 induce chromosomal instability (CIN), or chromosome missegregation during mitosis, which also causes DNA damage and can lead to profound genetic alterations in the host cell. Though these features are known to contribute to HPV-induced carcinogenesis, how this affects tumor cell response to DNA damaging treatments is not well understood. Here, we review how HPV induces DNA damage and activates the DNA damage response and how the HPV-induced CIN likely exacerbates this. We then discuss how this viral protein-mediated DNA damage may affect the efficacy of chemoradiation therapy.

**Abstract:**

High-risk human papillomaviruses (HPVs) are the main cause of cervical, oropharyngeal, and anogenital cancers, which are all treated with definitive chemoradiation therapy when locally advanced. HPV proteins are known to exploit the host DNA damage response to enable viral replication and the epithelial differentiation protocol. This has far-reaching consequences for the host genome, as the DNA damage response is critical for the maintenance of genomic stability. HPV+ cells therefore have increased DNA damage, leading to widespread genomic instability, a hallmark of cancer, which can contribute to tumorigenesis. Following transformation, high-risk HPV oncoproteins induce chromosomal instability, or chromosome missegregation during mitosis, which is associated with a further increase in DNA damage, particularly due to micronuclei and double-strand break formation. Thus, HPV induces significant DNA damage and activation of the DNA damage response in multiple contexts, which likely affects radiation sensitivity and efficacy. Here, we review how HPV activates the DNA damage response, how it induces chromosome missegregation and micronuclei formation, and discuss how these factors may affect radiation response. Understanding how HPV affects the DNA damage response in the context of radiation therapy may help determine potential mechanisms to improve therapeutic response.

## 1. Human Papillomavirus Genome and Lifecycle

Human Papillomaviruses (HPVs) are small 8 kb double-stranded DNA tumor viruses and are the most common sexually transmitted infection. Over 85% of men and women are estimated to be infected with HPV during their lifetime [1,2]. Although most infections are cleared naturally, HPV infection can persist in host cells. The alpha papillomaviruses have a mucosal tropism and are designated to be low-risk or high-risk depending on their capacity for malignant transformation, which largely depends on the affinity of oncogenes E6 and E7 for their targets: the tumor suppressors p53 and retinoblastoma (Rb), respectively. Over 200 types of HPVs have been identified and 40 infect the anogenital and oropharyngeal tracts. Of these, a subset (12 types) are considered oncogenic high-risk HPVs. The most common and frequently studied high-risk alpha-HPVs that cause cancer are HPV16 and HPV18 [3].

The HPV genome can be divided into three regions: the early region, the late region, and the long control region. The early region (genes denoted with “E”) encodes regulatory proteins for the virus, the late region (L) encodes the viral capsid proteins L1 and L2, and the long control region (also known as the upstream regulatory region) is responsible for regulating transcription and replication of the viral DNA [4]. The core HPV proteins, E1 and E2, are involved in viral DNA replication and amplification, while E1^E4, E4, E5, E6, E7, and E8^E2 [5] are involved in the viral life cycle and optimize the survival of the virus in its host by promoting immune evasion, cell growth, and inhibiting apoptosis (reviewed in [6]). 

HPV initially infects basal cells in stratified squamous epithelium to which its life cycle is intimately linked. Infection is thought to occur by binding to the basal epithelium due to microabrasions caused by physical or sexual contact and by the binding of L1 capsid protein to heparan sulfate proteoglycans, which are the main receptors of HPV [7]. Once the basal cell has been infected, the capsid is endocytosed and degraded, and the viral genome is coated with the L2 protein, which mediates trafficking from the trans-golgi network to the nucleus [8,9]. The virus then attaches to the mitotic chromatin of the host cell via an E2-mediated complex with BRD4 and TOPBP1 and remains tethered to the chromatin until the nuclear envelope reforms [10,11,12]. The virus is immediately amplified to 10–100 copies per cell in a process known as basal replication/amplification. As basal cells divide, the viral copy number is maintained through a process known as viral maintenance, a type of persistent infection. The virus is therefore able to reside in the nucleus which is imperative to its survival, as it depends entirely on the host cell machinery for viral DNA replication, although the polymerase repertoire that replicates HPV is not yet fully defined through the stages of the viral life cycle. This allows for hijacking of the host DNA damage response to support and promote its own DNA replication. 

Viral proteins E1 and E2 are essential for viral genome amplification and activate the DNA damage response, which recruits repair proteins to the site of viral replication (discussed in more detail below). This enables rapid and high-volume viral DNA synthesis resulting in hundreds of episomal copies per host cell, although the exact mechanisms are still not well understood. The canonical high-risk HPV oncoproteins E6 and E7 cause degradation of p53 and deregulate Rb, respectively, which stimulate cell cycle re-entry in otherwise quiescent upper differentiated epithelial layers to allow for viral genome amplification and delay epithelial differentiation by targeting PTPN14 [13]. Eventually one of these cells commits to differentiation, and the HPV genome is amplified from 10–100 copies per cell to thousands of copies per cell. The viral proteins L1 and L2 are then expressed, leading to the formation of functional viral particles and release from the outermost epithelial layer. In some cases, high-risk HPV can become integrated into the host cell’s DNA, signifying a dead end for the virus itself and causing grave consequences for the host. 

## 2. Causes of Carcinogenesis

Mucosotropic high-risk HPV causes 5% of cancers worldwide, including cervical, oropharyngeal, and anogenital carcinomas (anal, vaginal, vulvar, penile) [14]. Globally, cervical cancer is the fourth most prevalent cancer for women and is the second most common cause of years of life lost in women with cancer [15]. HPV-associated oropharyngeal cancer is rapidly rising in incidence in men in high-income countries and there are now more men with this diagnosis than women with cervical cancer in the USA [16]. The HPV vaccine has the potential to completely prevent the development of HPV-associated cancers, but only 58.5% of adolescents received the vaccine in 2021 [17] and rates are certainly not high enough for herd immunity [18]. In addition to this issue, there is a striking disparity in terms of screening for HPV, vaccination rates, and access to treatment that exist along racial, regional, and socioeconomic lines [19]. 

In general, HPV integration leads to increased and unbridled expression of E6 and E7, as they are now being driven by host promoters. E6 and E7 are both effective in immortalizing most cell types as they work in synchrony to promote growth and survival. However, E6 and E7 are necessary, but not sufficient, for carcinogenesis, as other somatic events in the host cell are required for this. E6 causes degradation of p53, leading to reduced apoptosis even in the presence of DNA damage and uncontrolled cell growth. This allows for the accumulation of damaged DNA, as well as genomic instability, which promotes tumorigenesis. E6 also encodes for a PDZ binding domain that binds nearly 20 proteins, including MAGI-1 and other tumor suppressors hDlg and hScrib [20,21]. The other main oncoprotein E7 targets tumor suppressor gene pRB, leading to the release of the E2F transcription factor promoting S-phase entry [22]. E7 also causes cell cycle deregulation, and together with abrogated cell cycle checkpoints mediated by reduced p53, allows for mitosis to proceed in the presence of mitotic errors leading to chromosomal instability (CIN). E6 and E7 alone and together also induce host DSBs [23] and drive immortalization through activation of the hTERT promoter [24]. In summary, E6 and E7 alter host cell physiology by decreasing growth arrest and cell death, increasing S-phase entry, abrogating mitotic checkpoints, and inducing DNA damage and replication stress, which leads to chromosomal instability and aneuploidy, all of which culminate in cellular transformation and tumorigenesis. 

Nearly all cases of locally advanced cervical, head and neck, and anal cancers are treated with definitive chemoradiation. HPV-associated cancers are generally more sensitive to radiation in all of these cancer types, but the difference is most striking in head and neck cancer [25,26,27]. Radiation induces double-stranded DNA breaks (DSBs), which initiate a profound DNA damage response with the recruitment of ataxia telangiectasia-mediated (ATM), ataxia telangiectasia and Rad3-related (ATR), and DNA-dependent protein kinase catalytic subunit (DNA-PKcs). Since HPV hijacks these pathways to promote viral replication, it may promote radiation sensitivity due to delayed or ineffective DNA damage repair (DDR). Alternatively, excessive activation of the DDR could lead to enhanced DNA damage repair following radiation, ultimately leading to radiation resistance. 

## 3. HPV Activates the DNA Damage Response

DNA damage within cells can be repaired through multiple mechanisms, including homology-directed recombination (HR), non-homologous end-joining (NHEJ), alternative end-joining (alt-EJ), mismatch repair, nucleotide excision repair, base excision repair, break-induced replication, and other DNA damage tolerance pathways, including translesion synthesis and repriming polymerases. The DDR network is characterized by the activation of three master kinases that are part of the phosphatidylinositol-3-kinase-related kinase (PIKK) family, including ATM, ATR, and DNA-PKcs [28,29]. DSBs activate the ATM, ATR, and DNA-PKcs kinases, while single-stranded DNA (ssDNA) breaks and replication stress activate the ATR kinase [29]. DSBs, such as those occurring after radiation exposure, are primarily repaired through HR and NHEJ [30]. NHEJ is facilitated by DNA-dependent protein kinase (DNA-PK), as well as ATM, and can occur throughout the entirety of the cell cycle [31]. HR is directed by ATM and ATR and only occurs in the S and G2 phases of the cell cycle, as it requires a homologous template for repair [32].

It has been well established that high-risk HPV can activate the DDR in order to promote viral genome amplification [33,34]. Both HPV proteins E1 and E2 have been shown to activate the DDR in HPV-expressing cells [35] (and personal communication with Dr. Iain Morgan, manuscript in revision). Because the viral genome is quickly amplified upon infection, the E1 and E2 origins of replication fire repeatedly resulting in a suggested “onion skin” pattern of replication that may contribute to structural DNA malformations and are a source of replication stress [36]. These aberrant structures are associated with increased DNA DSBs and can themselves initiate a DDR [37]. This is further supported by evidence that E1 and E2 localize to nuclear foci and are associated with phosphorylation of ATM and γH2AX, both markers of DSB formation and general replication stress [35]. E2 also increases DNA damage by forming a complex with BRD4 and TOPBP1 to gain access to host chromatin and the nucleus during initial infection in some HPV types [11,38]. The cohesin SMC1 and its binding partner, the DNA insulator and DNA looper CTCF, may be an alternative or complementary tethering mechanism besides BRD4 and E2 for viral maintenance [39]. In fact, SMC1 is constitutively activated in HPV+ cells and complexes with γH2AX and CHK2. Though E1 and E2 are best known for their involvement in viral replication and are thought to be largely absent in cancer cells following HPV integration into the host genome, viral genomes can persist as episomes in cancer cells alongside their integrated forms. This occurs most often in head and neck cancer [40], but there is evidence that both E1 and E2 are also expressed in cervical cancer cells and tissues [41,42,43], implying there may be some activation of the DDR by E1 and E2 in HPV+ cancers. 

Increased levels of topoisomerases are also present in HPV+ cells, likely due to the increase in replication stress induced by viral genome replication. HPV16 E7 increases the levels of topoisomerase 2β (TOP2β), which is associated with increased DSBs and seems to be necessary for HPV genome replication [44], again confirming the relationship between viral genome amplification and activation of the DDR. This replication stress-induced increase in topoisomerase activity activates the ATR pathway. Accordingly, the ATR signaling pathway was shown to be constitutively activated in HPV+ cells in the absence of exogenous DNA damaging agents [45]. HPV E7 also activates STAT-5, a regulator of innate immune signaling, which transcriptionally regulates TOPBP1, leading to further ATR activation [45]. There is evidence that HPV+ cells divert DNA damage repair proteins to the viral DNA at the expense of host DNA to selfishly ensure their own genome integrity [26]. This same study showed that HPV31+ cells have higher levels of small DNA fragments generated from DSBs and that the amount of DSBs correlates with the extent of viral genome amplification. Whether the virus selfishly utilizes host DDR proteins in cancer cells (when viral amplification is no longer occurring) is not known, though it is tempting to hypothesize that this could hamper efficient DNA damage repair after radiation leading to increased radiation sensitivity. Interestingly, the host proteins involved in enhancing viral replication, such as BRD4, cohesins, and CTCF, are all linked to radiation responses [46,47,48].

HPV both activates the ATM pathway and requires its activation for persistent replication [34]. HPV31+ cells have increased phosphorylation of ATM and its substrates, including CHK2, BRCA1, SMC1, and NBS1, compared to HPV- keratinocytes [26,34]. Virus-induced activation of ATM is necessary for viral genome amplification in differentiating cells, but not for the maintenance of viral episomes in undifferentiated keratinocytes [34]. Therefore, ATM effectors, such as γH2AX, 53BP1, Rad51, BRCA1, and members of the MRN complex, also localize to sites of viral replication at nuclear foci, and this is increased during differentiation-dependent amplification [33]. This could assist with either the maintenance of viral DNA integrity or resolving replication intermediates during viral DNA amplification. Indeed, ATM was demonstrated to resolve replication intermediates during SV40 infection, which has similarities to papillomaviruses [49]. Recent reports also indicate that R-loops formed by replication and transcription conflicts are critical for HPV pathogenesis and require HPV E6 [50]. 

**Figure 1:**
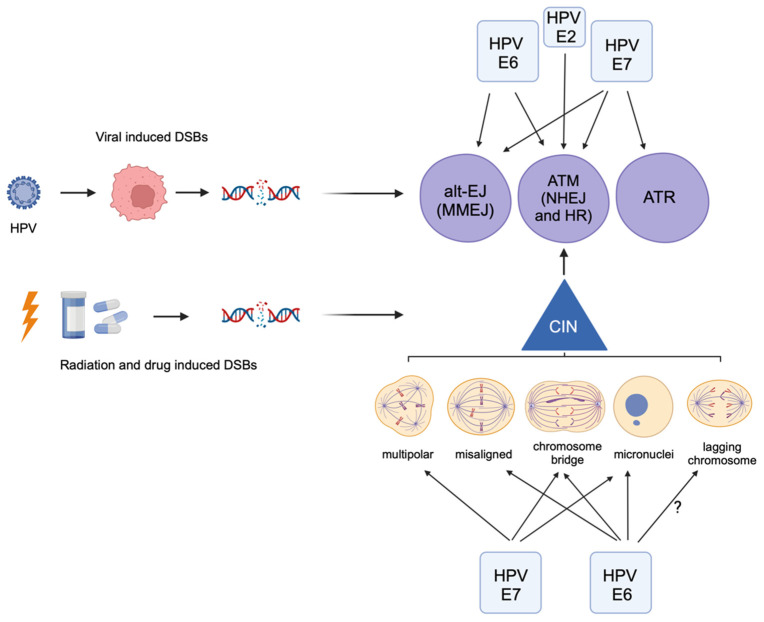
Human papillomavirus oncoproteins can activate the DNA damage response directly (**top**) or indirectly by inducing chromosomal instability (CIN, **bottom**). Viral-induced DSBs can also directly activate the DDR pathways. HPV E6 and E7 induce specific types of chromosomal instability, including misaligned and lagging chromosomes, chromosome bridges, multipolar spindles, and micronuclei formation, each of which can activate DDR pathways. Radiation and chemotherapeutic drugs induce DSBs, which also increase CIN and activate the DDR. Created with BioRender.com.

Importantly, E6 and E7 can induce DSBs and the ATM pathway in both host and viral DNA independently of viral replication, implying its activity in cancer cells (Figure 1). Keratinocytes expressing E6 and E7 had increased DNA damage as indicated by γH2AX foci, which was associated with an increase in PARP expression [51,52] and both upregulated pathways involved in DNA repair (both NHEJ and HR pathways), including BRCA and PARP1 genes, as well as blocking other DNA damage pathways, such as translesion synthesis [53,54]. E7 itself may also be increasing DSBs by inactivating Rb, as upregulation of E2F1 induces an increase in DSBs in cells [55]. This is consistent with the fact that activation of ATM and ATR and several downstream factors by E7 is dependent on the E7 Rb-binding domain [56]. RNF168 in host cells is critical for the DDR and DNA DSB repair and is downstream of γH2AX signaling. It is also required for productive viral replication; however, E7 was found to decrease the levels of RNF168 recruited to DSBs, ultimately affecting the DNA pathway repair choice and directing it toward HR. Both HPV+ cervical and head and neck cancer cells express high levels of RNF168 mRNA, which is likely an adaptation to chronic E7-mediated RNF168 sequestration [57]. HPV+ anal and cervical tumors were found to have enlarged nuclear 53BP1 bodies and high levels of RNF168, which were not present in HPV- tumors. This was associated with increased NHEJ and HR and radiation resistance in vitro [58]. However, another group found that the E7-mediated increase in p16 leads to decreased HR activity [59], which is consistent with the enhanced radiation sensitivity observed clinically. 

There is ample evidence that HPV can activate the DDR in vitro, and this appears to also be true in HPV+ tumors from patients. The expression of DNA repair factors is increased in high-grade cervical intraepithelial lesions compared to low-grade [60]. This is also true in HPV+ oropharyngeal cancers compared to HPV- head and neck cancers. Specifically, HPV+ head and neck cancers have increased levels of pCHK1, FANCD2, BRCA1, RAD51, and γH2AX foci, implying the presence of increased DSBs and activation of the ATR pathway in human tumors [61]. Another study confirmed that HPV+ head and neck tumors have higher expression of DNA repair genes across all DDR pathways, including higher BRCA1 and Rad51 protein levels than HPV- head and neck tumors [62]. Upregulated DDR proteins may serve as effective therapeutic targets. Inhibition of ATR impaired HPV DNA amplification, caused DNA damage and apoptosis in an E7-dependent manner, and sensitized cervical cancer cells to cisplatin [63]. However, ATR inhibition did not increase radiation sensitivity in HPV+ compared to HPV- head and neck cancer cells [64], implying there may be other DDR pathways that can substitute for the loss of ATR. In general, there seems to be potential in targeting the DDR to specifically induce HPV+ cell death.

Not only can HPV activate the DDR, but it can also hamper the efficiency of DNA damage repair, which has significant implications for the host cell. HPV+ cell lines and HPV16 E6 and E6+E7 expressing cells have a delay in DDR following radiation [52,65]. This may indicate that the viral upregulation of DDR proteins impairs their utilization. HPV16 E6 was shown to repress HR, which is due to the initiation of HR during G1, as there is no sister chromatid present to act as a template for the repair and mislocalization of Rad51 complexes [52]. Additionally, HPV+ head and neck cancer cell lines have an impaired ATM-mediated DNA damage response compared to HPV- cells despite having functional ATM [66]. Thus, there is evidence that both ATM and ATR pathways are compromised in HPV+ cells, which may explain their enhanced radiation sensitivity. HPV16 E6 was shown to degrade the translesion synthesis (TLS) pathway polymerase eta (POLH) which leads to increased replication fork collapse and sensitivity to treatments that induce replication stress, such as cisplatin [54]. Conversely, HPV E7 induces p63 expression to facilitate a DDR, allowing for progression through the cell cycle and continued cellular growth after exposure to ionizing radiation [67]. This may be associated with radiation resistance. Future studies will need to investigate the importance of other TLS polymerases and DDR pathways in HPV-associated cancers and how these may affect chemoradiotherapy response. 

If HPV+ tumors have deficient HR repair, it is possible that they exhibit a “BRCAness” phenotype that would imply sensitivity to PARP or CHK1 inhibition. Overexpression of p16, which is mediated by E7-induced Rb inhibition, leads to suppression of HR and increases sensitivity to the PARP inhibitor Olaparib [59]. Furthermore, three out of nine primary HPV+ cervical cancer cell lines were highly sensitive to PARP inhibition [68]. However, other studies have shown that there is no difference in sensitivity to PARP inhibition between HPV+ and HPV- head and neck cancer cells [66,69]. Further biomarker studies regarding other hallmarks of BRCAness to predict responses to PARP inhibition in HPV+ cancers need to be performed to better guide future therapeutic interventions. 

## 4. HPV Induces Chromosomal Instability, Which Can Lead to Further DNA Damage

During mitosis, the kinetochores of each sister chromatid attach to microtubules emanating from opposite spindle poles. Once all kinetochores are properly attached to microtubules, the spindle assembly checkpoint is satisfied, and anaphase leads to the separation of the sister chromatids yielding two genetically identical daughter cells. Approximately 50% of cancer cells have aberrant mitosis, or chromosomal instability (CIN), which is the continued missegregation of whole chromosomes or chromosome arms or fragments over successive mitotic divisions. Mitotic errors include misaligned or lagging chromosomes, chromosome bridges, or multipolar spindles, which are discussed in more detail below (and reviewed in [70]).

HPV16 oncogenes E6 and E7 are known to induce CIN and have been implicated in the formation of misaligned, lagging, and bridge chromosomes [51,71]. High-risk HPV has also been shown to induce centrosome amplification, resulting in multipolar spindles and multipolar divisions [51,72] (and reviewed in [73]). Centrosome amplification and spindle pole multipolarity cause CIN directly by promoting multipolar divisions, which is often lethal [74,75]. This can also cause CIN in the form of lagging chromosomes, although this has not been studied in the context of HPV [74]. Cosper et al. recently showed that HPV16 E6 causes the specific degradation of centromere protein E (CENP-E), which stabilizes microtubule capture by kinetochores and is required for chromosome alignment at metaphase. This E6-induced degradation of CENP-E results in chromosomes misaligned at the spindle pole and was found in HPV+ cell lines, HPV+ patient-derived xenografts, as well as HPV+ head and neck cancers from patients [71]. HPV16 E6 and E7 also induce chromosome bridges, which is likely due to increased DNA damage with incorrect repair, as well as telomere erosion [51,76]. However, others have shown that HPV E6 and E7 induce telomerase activity [77,78] by epigenetically or directly regulating the hTERT promoter, or by post-transcriptional regulation of the promoter (reviewed in [79]). Perhaps there is a balance between telomere erosion and elongation in human tumors.

**Figure 2:**
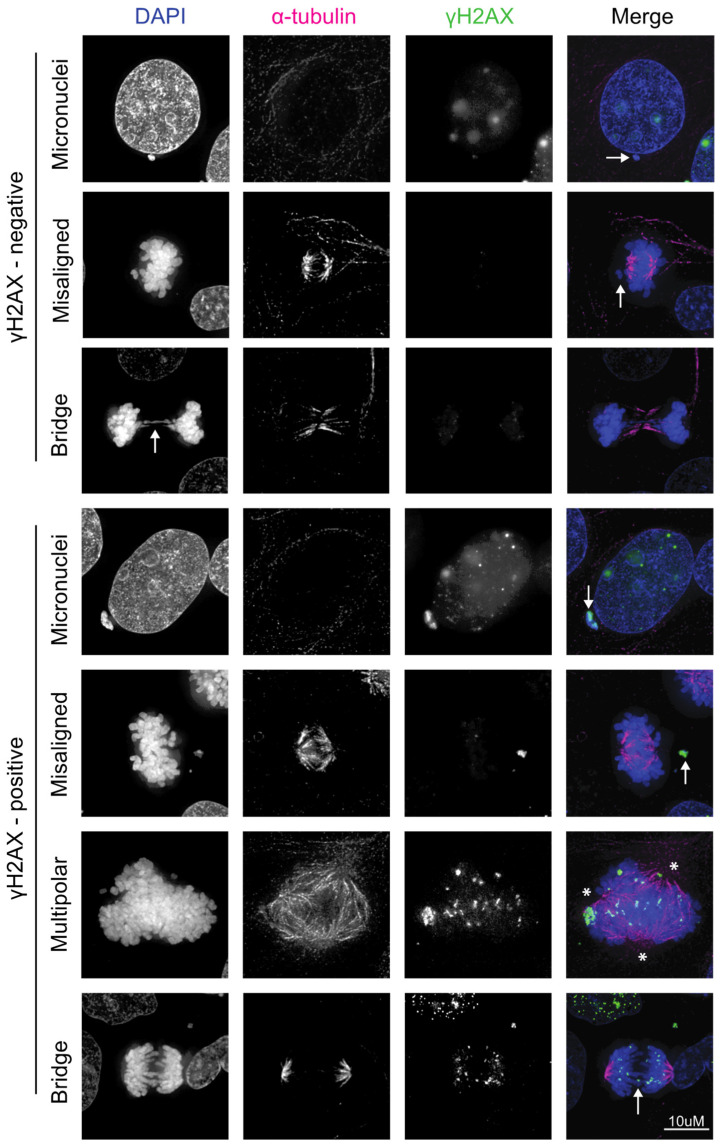
Representative immunofluorescent images revealing how chromosome missegregation can be associated with DNA damage in HPV+ cells. Images are of untreated 93-VU-147T (HPV16+) head and neck cancer cells undergoing mitosis with evidence of chromosomal instability (CIN) in the form of micronuclei, misaligned chromosomes, chromosome bridges (indicated by arrows), or multipolar spindles (spindles denoted by asterisks). Recognition of DSBs by ATM leads to the phosphorylation of the histone H2AX yielding γH2AX. The top three panels represent examples of interphase or mitotic cells with CIN that are not associated with DNA damage, while the bottom four panels represent examples of CIN that are associated with DSBs and γH2AX signaling. Thus, CIN is not always associated with DNA damage. The 93-VU-147T cells were fixed with paraformaldehyde, incubated with anti-tubulin or anti-γH2AX antibodies, and counterstained with DAPI. (Blue, DAPI; pink, alpha-tubulin; green, γH2AX). All images were acquired using a Nikon Eclipse Ti2-E (Nikon, Yokohama, Japan) inverted fluorescence microscope with a 100×/1.4 numerical aperture oil objective. Images are maximum projections of 0.2 μm z-stacks that have been deconvolved.

While it is clear that HPV induces many types of CIN, it is not known whether HPV-induced CIN is associated with increased DNA damage or an altered DNA damage response. There are, however, many lines of evidence that CIN induced by other mechanisms causes DNA damage, implying that this may also be the case in HPV-induced CIN (Figure 1 and Figure 2). Chromosome bridges (reviewed in [80]) occur when DNA is stretched between opposite spindle poles due to the presence of dicentric chromosomes, which can be caused by radiation or telomere crisis [81], or defects in DNA decatenation or sister chromatid cohesion [82,83]. Bridges are perhaps the best example of CIN directly causing DNA damage because they can rupture after mitosis in a process requiring actomyosin ring contractile forces [84,85,86]. This can initiate a breakage-fusion-bridge (BFB) cycle, where broken ends from different chromosomes join producing another dicentric chromosome, which is destined to form a bridge and perpetuate DNA damage. Evidence of BFB cycles has been found in cervical cancer cells [87]. The 3′ exonuclease TREX1 localizes to chromatin bridges and generates ssDNA that ultimately joins the daughter nucleus. The remnants of these broken bridges activate the DNA damage response as they stain positive for γH2AX, 53BP1, and MRE11 [85]. Analysis of daughter cells following bridge breakage revealed reciprocal chromosome segment gain and loss, as well as evidence of DNA fragmentation and rearrangements from the ligation of these fragments [86]. The ends of broken bridges further activate the DDR, as they erroneously undergo DNA replication during mitosis [86]. It is important to note that not all bridges break during mitosis and many persist into the next interphase as intercellular bridges [85,88]. This is theorized to be due to alterations in K-fiber kinetics during anaphase to avoid breaking during mitosis [88]. 

Chromosome missegregation causes DNA damage with increased γH2AX, 53BP1 recruitment, and activation of ATM on missegregated chromosomes [84] (Figure 2). This mostly occurs on chromosomes trapped in the cleavage furrow as only 10% of missegregated chromosomes outside of this region had evidence of DNA damage. Chromatids with unattached kinetochores activate the spindle assembly checkpoint as the cell attempts to correct this error, resulting in mitotic arrest. This, and other activators of the spindle assembly checkpoint, results in prolonged mitosis, which itself leads to DNA damage [89]. Anal tissue positive for high-risk HPV subtypes has significantly higher levels of DNA damage during mitosis compared to control tissue, and this increases with pathological grade. This was shown to be due to E7’s ability to abrogate the G2 checkpoint and promote mitotic entry in the presence of DNA damage [90]. HPV is therefore associated with DNA damage during mitosis, which may be due to chromosome missegregation events, leading to a further upregulation of the DNA damage response. However, whether this functionally affects the DDR and, therefore, radiation response remains to be determined. 

Missegregated chromosomes often end up in micronuclei, which are distinct membrane-bound compartments containing DNA that are separate from the main nucleus. Misaligned, lagging, and bridge chromosomes can all lead to the formation of micronuclei, though lagging and bridge chromosomes are the most common sources [91]. It has been reported that bridges do not result in micronuclei formation during the initial abnormal mitosis [85] but over half of the cells undergoing the subsequent division formed micronuclei [86]. Missegregation of a whole chromosome with micronucleus formation does not cause DNA damage initially, but DNA damage is significantly increased during the subsequent G2 phase due to defective DNA replication during the S phase [92]. This results in pulverization of chromosomes that cause extensive genomic rearrangements in a process known as chromothripsis [86,92,93], which is a common fate of DNA in micronuclei. Chromothripsis is characterized by massive intrachromosomal rearrangements in a single chromosome or chromosome arm. This damaged DNA can re-incorporate into the main nucleus during the next mitosis [92], leading to genomic heterogeneity with the possible consequence of tumor cell genome evolution. Additionally, micronuclear envelopes are prone to rupture partially due to lack of lamin B1, which further increases DNA damage and releases cytosolic DNA, resulting in activation of an inflammatory signaling cascade [94,95].

While micronuclei themselves can cause DNA damage, DNA damage due to abnormal DNA replication and repair or DNA damaging agents, such as radiation, can lead to the formation of micronuclei. For example, disruption of Fanconi anemia (FA) repair proteins and the DNA damage response mediator proteins MDC1 and TOPB1 lead to increased micronuclei formation [96,97]. FANCD2, a member of the FA complex, not only binds high-risk HPVs but is required for episomal maintenance [98]. Thus, episomal HPV in tumors may dilute FANCD2 from the host DNA, resulting in repair defects and micronuclei. 

Ionizing radiation is one of the most well-known inducers of micronuclei and was observed over 60 years ago [99]. In fact, micronuclei are quantified in the cytokinesis-block micronucleus assay, which is a well-established sign of prior radiation exposure [100]. This is due to radiation-induced CIN and the formation of acentric fragments, which are chromosome fragments that lack a centromere. These are unable to attach to microtubules as they lack a kinetochore, are missegregated, and often end up in micronuclei [101], though chromosomes with a centromere can also form micronuclei when missegregated. NHEJ is the major DSB repair pathway following radiation-induced chromosome missegregation and often leads to significant chromosomal rearrangements [102]. Micronuclei have defective DNA damage repair, where components fail to be recruited, resulting in a slow resolution of γH2AX foci after irradiation [92,103]. Thus, radiation induces DNA damage beyond the initial dsDNA breaks by inducing many types of CIN, which can result in chromosomal rearrangements and micronuclei, which perpetuate DNA damage and hamper DNA repair. 

HPV16 E6 and E7 expression increase micronuclei frequency [104] (Figure 2). Accordingly, micronuclei are more prevalent in HPV+ cervical smears than HPV- [105] and there is a significant association between HPV infection and micronuclei frequency [106,107]. Cervical smears from women with cervical intraepithelial neoplasia grade I had significantly more micronuclei than normal or HPV- cervical cells and the extent of micronuclei correlated with viral load [107]. Furthermore, micronuclei increase with increasing grade of dysplasia in cervical smears, are highest in invasive squamous cell carcinoma [108] and are associated with the persistence of intraepithelial neoplasia [109]. This was corroborated in a meta-analysis of 21 studies correlating the incidence of micronuclei with the grade of cervical dysplasia [110]. The Beta genus of HPV is suspected to play a role in the promotion of squamous skin cancers and it was found that HPV8 E6 also caused increased anaphase bridges and micronuclei, and induced chromothripsis [111]. Thus, induction of CIN resulting in micronuclei may be a feature of many different types of HPV and is likely causing increased DNA damage, which contributes to carcinogenesis. How this DNA damage affects radiation response is not well understood, but it has been shown that tumor cells with high levels of CIN are more sensitive to radiation [112] (and reviewed in [113]).

## 5. HPV+ Cancers Use the Alternative End-Joining DNA Repair Pathway

Alternative end-joining (alt-EJ), also termed microhomology-mediated end-joining (MMEJ [114]) and polymerase theta mediated end-joining (TMEJ [115]), will be referred to as alt-EJ henceforth. Alt-EJ is defined as a DSB repair that is distinct from NHEJ, acts on DNA ends with resection-dependent 3′ ssDNA overhangs, and produces repair products with large deletions (~30–200 bp) that extend to microhomologies [115,116,117]. It is a highly error-prone pathway that promotes inter- and intra-chromosome rearrangements related to DNA deletions by using sequence microhomology to recombine broken DNA ends [114,117,118,119,120]. Additionally, alt-EJ is similar to HR as both occur only in the S and G2- phases of the cell cycle and each uses the same DNA resection factors, including Mre11 and CtlP, to promote the formation of 3′ ssDNA overhangs at DSBs [32,117,118,121]. 

Polymerase theta (Polθ) has been identified as an essential protein for DNA synthesis and repair, DNA end-joining, and reestablishing replication following replication fork collapse in the alt-EJ pathway [115,117,122,123,124]. Alt-EJ activation is regulated through DSB recognition and is rarely used under normal conditions. However, when resection is misregulated or NHEJ is compromised, Polθ is engaged in a larger fraction of repair, thereby initiating alt-EJ [115,125,126,127,128,129]. Its ability to synthesize DNA in vitro is low and error-prone, while also inducing substitutions, insertions, and deletions, all at similar rates [115].

HPV+ head and neck cancer cells have been found to suppress HR and increase the use of the alt-EJ pathway, which is partially due to a lack of responsiveness to TGF-β signaling [130]. Analysis of the TCGA head and neck cancer cohort confirmed that HPV+ cancers have low expression of TGF-β target genes and upregulated alt-EJ genes compared to HPV- cancers [131]. TGF-β signaling is therefore inversely correlated with the use of the alt-EJ pathway [130,131]. Additionally, genomic sites of HPV integration in cervical and oropharyngeal cancers are highly enriched for microhomology, a defining characteristic of alt-EJ [114,132,133]. HPV8 (β-HPV) E6 also promotes alt-EJ for DSB repair [134] suggesting a conserved viral mechanism to ensure DNA repair, which is likely contributing to genomic instability. Further mechanistic studies revealed that E7 suppresses NHEJ and promotes alt-EJ, matching human-level evidence in HPV-associated cancer genomes [114]. 

Alt-EJ may play an important role in dictating radiosensitivity as it is highly error prone and, therefore, more likely leads to DSB repair incompatible with viability, or further mitosis [114,119,120]. Indeed, upregulation of this altered DDR pathway predicted better response to DNA damaging therapy, including radiotherapy in multiple cancer types, leading to improved patient outcomes [131]. Thus, utilization of alternative DSB repair pathways, such as alt-EJ, is associated with a reduced accuracy of DNA repair, which may contribute to enhanced radiation-induced cell death. Crucially, Polθ inhibitors have been recently described and are currently in the clinic [135]. Future studies need to clarify HPV+ cancer response to these inhibitors in the presence and absence of radiation.

## 6. Conclusions

HPV infection is nearly ubiquitous in both men and women and can cause cancers of the head, neck, and anogenital tracts. HPV oncoproteins induce DNA damage and activate the DNA damage response both during viral replication and after viral persistence in dysplastic and malignant tissue. HPV also induces many types of CIN, which are often associated with significant DNA damage and activation of the DDR. We think these two independent mechanisms of inducing DNA damage may overwhelm the host DDR, which could reduce the efficiency of DNA damage repair following chemoradiation. For example, viral quenching of the DDR proteins could be a mechanism for the increased radiation sensitivity of HPV+ cells that have been observed in both pre-clinical and clinical studies. Our laboratory is currently studying how specific types of CIN modulate radiation response as we aim to provide more personalized therapeutics. Further understanding of how activation of the DDR in HPV+ tumors affects radiation response is vital for the discovery of novel therapeutic approaches.
